# Evacuation in Buildings Based on BIM: Taking a Fire in a University Library as an Example

**DOI:** 10.3390/ijerph192316254

**Published:** 2022-12-05

**Authors:** Haotian Zheng, Shuchuan Zhang, Junqi Zhu, Ziyan Zhu, Xin Fang

**Affiliations:** 1 School of Safety Science and Engineering, Anhui University of Science and Technology, Huainan 232000, China; 2 School of Economics and Management, Anhui University of Science and Technology, Huainan 232000, China

**Keywords:** personnel evacuation, BIM, pathfinder, the most unfavorable principle, evacuation efficiency

## Abstract

As a typical public place, a university library has a large collection of books with heavy fire load, dense population, and large flow of people. The situation of safe evacuation in case of fire is very serious. This study utilizes Revit, Pyrosim, and Pathfinder software to research evacuation of a university library. First, a Building Information Modeling (BIM) is constructed based on Revit software in 1:1 scale. Second, the evacuation passage with the highest utilization rate was determined through Pathfinder software. According to the “most unfavorable principle,” the location near it was assumed to be where the fire occurred. Pyrosim software was used to determine the smoke spread, visibility, CO concentration, temperature, and other conditions at each stairway exit in case of fire. Finally, the evacuation situation is compared with that after man-made route planning. The results indicate that evacuation exits 1#, 7#, 13#, 19#, and 23# have the highest utilization rate. The safe evacuation time was 739.275 s, which was shortened to 638.025 s after man-made route planning, a 13.67% increase in evacuation efficiency. Evacuation efficiency can be significantly improved by increasing broadcast guidelines, adding signs, widening staircases, and other optimization suggestions, which can provide reference for the study of evacuation effects in public places and the improvement of the layout of public facilities.

## 1. Introduction

Due to rapid economic development, many countries have built large public buildings [1]. However, in case of fire and other emergencies, it is difficult to escape from such a structure that gathers a large number of people. Therefore, in recent years, how to evacuate safely, effectively, and quickly has become a very hot research topic. As one of the important places for teachers and students to study, the university library is a typical public place with major hazards [2]. The scale of university libraries tends to expand continuously, the functions tend to be diversified, the collection of books is large, the fire load is large, and personnel are dense. These characteristics directly lead to the increasing possibility and severity of fire. In case of fire, it is very easy to sustain heavy casualties and property losses. On 2 March 2003, at least 35 people were injured when a fire broke out in Egypt’s famous Library of Alexandria due to an electrical short circuit. On 3 September 2004, a fire broke out in the Anna Amaria Library in Weimar, Germany, destroying 30,000 rare books, which was called a “cultural disaster for Germany and a grave loss for the world history”. On 30 January 2015, a fire broke out in the library of the Institute of Social Science Information in Moscow, Russia. The roof of about 1000 square meters collapsed, and 15% of the books were burned, including a large number of ancient books, documents of the United Nations and other precious materials. It is known as the Chernobyl event of the Russian scientific community. Therefore, the research on the safe evacuation of library personnel is of great significance [3].

At present, scholars at home and abroad have done a lot of research on library fire and personnel evacuation. Li Mingxin et al. simulated the fire spread and personnel evacuation of a university library in Nanjing by Pyrosim and Pathfinder and evaluated the fire safety of the library by comparing the risk time and evacuation time [4]. Hu Jiangwen et al. simulated the full evacuation of the library with Pathfinder and found that there was imbalance in the use of evacuation stairs, incoordination in the loading quantity of the northern and southern regions, and the difference in evacuation capacity between the two regions [5]. Mufeng Xiao adopted Pathfinder software to model a library project on the main campus of Liaoning Technical University and found that the bottleneck areas of evacuation were indoor corridors and exits of evacuation stairs, and the number of piles and location planning of items in the reading area greatly affected the evacuation time [6]. Guo et al. took the comprehensive library of a university as the research object, set three fire places through Pyrosim software, analyzed the spread of smoke in the library from three aspects of temperature (CO volume, fraction, and visibility), and obtained ASET under different working conditions [7]. Ruggiero Lovreglio et al. provided new pre-evacuation data collected in four evacuations of the same university library and illustrated that unplanned evacuations had higher pre-evacuation times [8]. At present, scholars at home and abroad have conducted extensive research on the problem of evacuation in public places, mainly focusing on performance-based fire protection design [9,10,11], planning escape routes based on algorithm models [12,13,14,15], the impact of fire smoke spread on evacuation [16,17,18,19], changes in psychological characteristics and behavior of personnel during evacuation [20,21,22,23], and so on. In terms of performance-based fire protection design, Khorasani et al. provide a comparative analysis of the fire performance of a floor system designed following prescriptive- and performance-based approaches [24]. Zhang et al. discussed an integrated fire structure simulation model for performance-based design. This was successfully applied to simulate the fire thermal structure behavior of real scale steel beams in local fire tests [25]. Bisby et al. summarized the fire protection design of concrete members strengthened with fiber-reinforced polymer [26]. In an experimental study, Zehfuss et al. investigated the thermal properties of selected fire-resistant materials (FPM) for steel members exposed to natural fire [27]. In terms of planning escape path based on an algorithm model, Zheng et al. proposed an invasive weed optimization algorithm, which has obvious advantages in convergence speed and evacuation time of crowd emergency evacuation simulation and effectively improves crowd emergency evacuation performance [28]. To improve the efficiency of emergency evacuation of subway passengers, Wang et al. suggested the concept of “guiding and platoon partition” and an intelligent algorithm of guiding and shunting for emergency evacuation of subway stations [29]. Zhu et al. adopted the lasso regression method to consider the road factors in pedestrian speed. Based on the improved 3D Dijkstra algorithm, they proposed an evacuation route optimization method under different flood risk levels [30]. Liu et al. expressed the intelligent evacuation route planning problem of emergencies (including natural and human resource disasters and COVID-19 pandemic) as the maximum flow problem and proposed a binary search algorithm based on the maximum flow algorithm, which is a community-oriented intelligent optimization evacuation route planning algorithm [31]. Wang proposed an improved path planning algorithm based on BEME (Multi-Exit Balanced Evacuation) on the basis of analyzing the factors affecting crowd path selection. This algorithm can further shorten the global evacuation distance of symmetrical evacuation scenarios, effectively balance the number of pedestrians at each exit, and reduce the evacuation time [32]. In terms of the impact of fire smoke spread law on evacuation, through numerical simulation, Zheng et al. examined the evacuation dynamics under fire and smoke, conducted in-depth analysis of the impact of personnel density, fire location, exit width, fire spread rate, and smoke diffusion rate on evacuation efficiency, and proposed an extended field model [33]. Through a series of numerical simulations, Li et al. determined the influence of fire heat release rate and fire spacing on fire plume generation in tunnels and proposed a prediction model considering heat release rate and burner edge spacing [34]. Using computational fluid dynamics and numerical simulation methods, Wang et al. investigated the smoke diffusion process under different ceiling heights [35]. Huang et al. established the process of fire growth and smoke diffusion in large space buildings through fire dynamics simulation and discussed the alarm process of a linear infrared beam smoke fire detector and an inspiratory smoke detection system [36]. In terms of changes in psychological characteristics and behavior of personnel during evacuation, Xue et al. proposed a new pedestrian model, which integrates fuzzy logic theory into a multi-agent system to solve the cognitive behavior of introducing uncertainty and imprecision in the decision-making process [37]. Zhao et al. built an improved SIRS model of passenger panic propagation in subway emergency based on passenger density, characteristics of confined space in subway cars, and passenger psychological factors [38]. Based on questionnaire data, Sieben et al. studied the collective phenomenon of pedestrian dynamics during gathering and dispersion [39].

However, the current research on the evacuation of people in public places mainly focuses on the microcosmic aspects, such as the psychological changes and behavioral characteristics of people in the evacuation process, the best choice of routes in the evacuation process, and so on, and there is less research on the overall understanding of evacuation in public buildings from the macro level in the case of fire. It is of great significance to investigate the whole building from the macro level. In the initial design of the building, it can be changed according to the feedback of the simulation results, which can effectively reduce the cost [40]. For existing buildings, the reconstruction based on the simulation results can ensure the safety of people’s lives and property [41]. Therefore, this study takes the typical representative university library in the public place as an example. The BIM model lightweight technology was adopted. Pyrosim software is used to simulate the smoke spread, visibility, CO concentration, and temperature of the library fire. Pathfinder software is used to simulate the evacuation situation, observe the evacuation path, calculate the evacuation time, and propose an effective emergency evacuation plan. A reasonable and effective solution to the problem of evacuation in public places is also explored, and a strategy for the design and transformation of public places at the initial stage of architectural design or completed buildings is provided.

## 2. Building Model Construction and Personnel Parameter Setting

### 2.1. Library Model Parameter Setting

**Figure 1:**
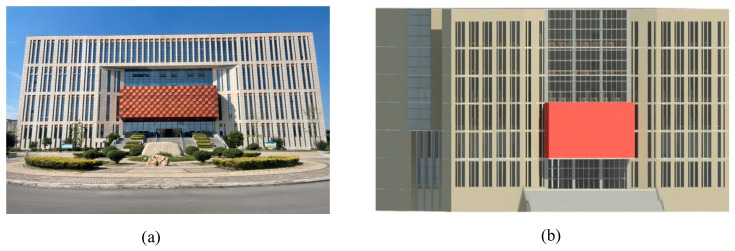
University library. (**a**) Front view of library. (**b**) Building Information Model.

The university library has a building area of 48,000 m^2^, a floor height of 3.8 m, a length of 104.8 m from east to west, a width of 48 m from south to north, and an area of 4154 m^2^. As of the end of 2021, the library will have 1.87 million books. The library has a total of seven floors (one underground, six above ground). The underground floor is mainly an underground garage, while the six floors above ground are equipped with a library, newspaper reading room, multimedia reading room, self-study room, conference room, and so on, which can meet the needs of teachers and students for borrowing books, meeting, and learning. Considering the main distribution and concentration of personnel, this study mainly focuses on floors one to six of the library. The building information model of the library is constructed using BIM technology in 1:1 scale, as shown in Figure 1.

Since the simulation scenario is a fire, the use of elevators is not considered [42]. There are two emergency exits on the first floor, marked as A and B. There are six evacuation stairs on each floor from the second to the fourth floor, whose numbers are marked as 1–18#. There are four evacuation stairs on each floor from the fifth to the sixth floor, marked as 19–26#. The specific numbers of each set of stairs are presented in Table 1.

### 2.2. Personnel Behavior Characteristics and Parameter Selection

The behavior characteristics of personnel in case of fire are important factors affecting evacuation which directly determine the evacuation time and efficiency [43]. Therefore, the accuracy and authenticity of evacuation simulation results are determined by the parameter selection based on the characteristics of human behavior [44]. The problem of personnel evacuation in university libraries has the following three characteristics [45,46,47]:

(1) It is difficult to evacuate due to the gathering of people. The library provides a large number of reading seats for the public to study, and each reading room has a large layout area; hence, the density of people is large, and very crowded, especially during exam week, which adds difficulties regarding rapid and safe evacuation.
(2) The personnel composition is relatively simple. Most of the staff in and out of university libraries are students, so the population is simply divided into adult men and adult women. Gender differences lead to different behaviors of men and women in the face of fire. For example, men will make more rational judgments and actively help other people, while women have weak psychological endurance and they will first consider their own choice of evacuation route.
(3) Strong action ability and fast escape. Adult men and women have strong mobility. Most have experienced evacuation drills and training. Once a fire occurs, people evacuate quickly and efficiently.

According to official statistics, there are 5185 seats in the university library, and the ratio of male students to female students is 2.2:1. The most unfavorable situation is considered in this study. First, it is assumed that there is no vacant space in the library, that is, there are people sitting in all positions. Second, the proportion of men and women is 2:1. Owing to the weak evacuation ability of women, the increase in the number of women leads to an increase in evacuation time. According to the number of seats in the reading rooms on each floor of the library, the number of simulation personnel is set to 5185 in total. The number of people that can be accommodated from the first floor to the sixth floor is 335, 700, 672, 632, 1420, and 1426, respectively. The basic information of each floor is presented in Table 2.

According to the literature [48], the speed of men in an emergency is 1.5 m/s, and that of women is 85% of that of men, that is, 1.28 m/s. According to the literature [49], the average shoulder width of men is 50 cm and that of women is 45 cm. Personnel parameter settings are reported in Table 3.

### 2.3. Numerical Simulation Software

**Figure 2:**
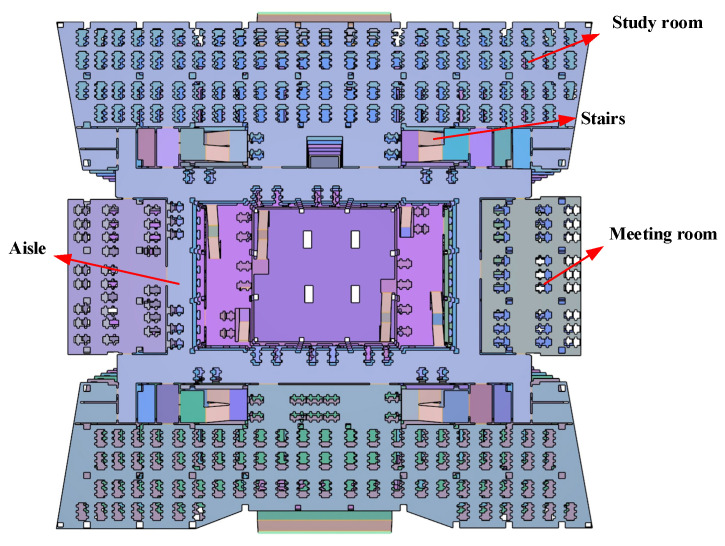
Building library internal model based on Revit.

**Figure 3:**
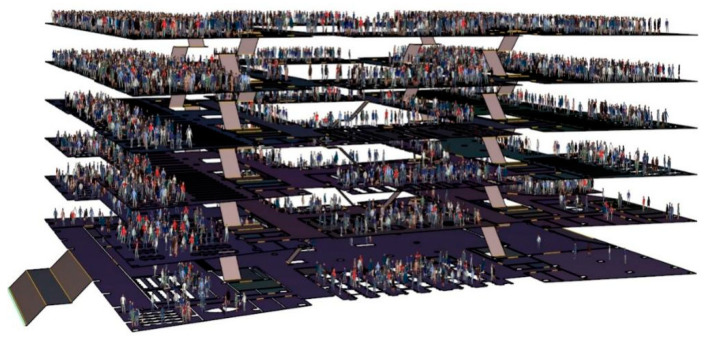
Library model of adding personnel.

Revit, Pyrosim, and Pathfinder software are used to conduct targeted research on a university library. Revit is one of the most widely used software packages in the BIM system of China’s construction industry and can help architectural designers design, build, and maintain buildings with better quality and higher energy efficiency [50]. The BIM information model of the library based on the Revit platform is illustrated in Figure 2. Developed by Thunderhead Engineering Company in the United States, Pathfinder is a simulator based on human movement and is widely used in fire evacuation simulation [51]. The combination of the BIM building information and Pathfinder evacuation simulation models can not only improve simulation efficiency, but also build a real and accurate simulation scene, as illustrated in Figure 3. Pyrosim is the most distinctive fire dynamics simulator based on the fire smoke movement field model developed by the National Institute of Standards and Technology (NIST) [52]. Its core technology is based on FDS technology, which has been improved. By dividing the space into several grids, each grid is given a unified parameter value, which can greatly improve the simulation accuracy [53].

**Figure 4:**
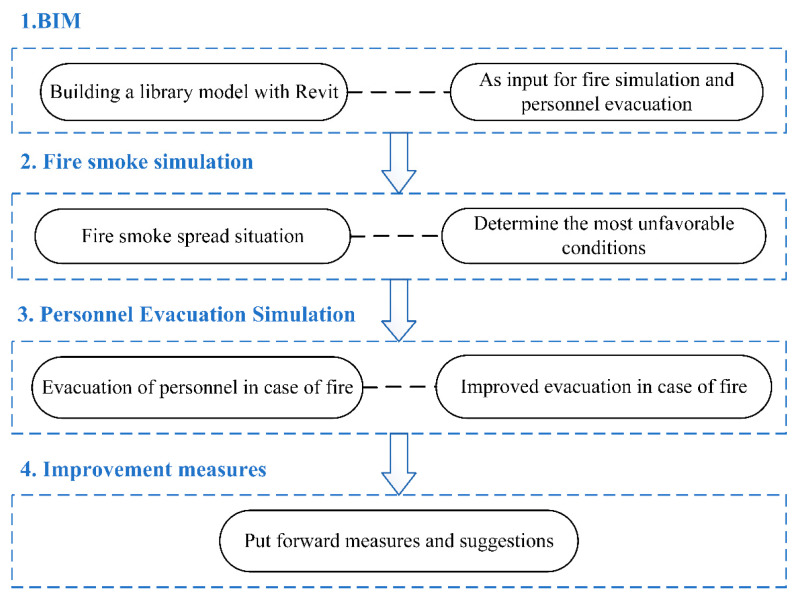
Research roadmap.

First, the author conducted field research on the library, established a library model according to the 1:1 ratio, considered the most unfavorable conditions, set the seat utilization rate as 100% and the male and female ratio as 2:1, simulated the evacuation situation, and obtained the utilization rate of each set of stairs and the total evacuation time. The location near the highest utilization rate of stairs was set as the fire source point, and Pyrosim was used to simulate the fire smoke. Considering the evacuation situation in case of fire, the utilization rate of each set of stairs and the total evacuation time can be obtained. Finally, this study assumed that the escape path is planned with signs and language guidance and that the modified evacuation time and stair utilization rate can be obtained. The overall research idea is presented in Figure 4.

## 3. Analysis of Simulation Results

### 3.1. Evacuation Passage of the Library Is Normal

**Figure 5:**
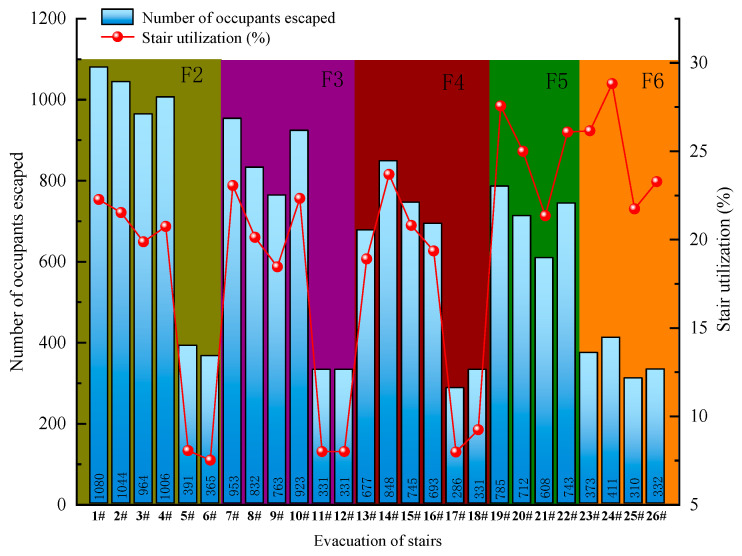
Evacuation under normal conditions.

A university library model is first established based on Revit software in 1:1 ratio, and Pathfinder is used to simulate the evacuation situation. Under the most unfavorable conditions, the seat utilization rate is set to 100%, and there are 5185 people in the library. The simulation results of Pathfinder software reveal that it takes 627 s for all people to complete the evacuation under normal conditions, including 2715 and 2470 people from the south and north doors, respectively. Evacuation channel utilization rate refers to the ratio of the number of evacuees from a certain channel to the total number of evacuees from all channels on the floor [54]. The total number of evacuees from all passageways on the second floor is 4850, and the number of evacuees in exit 1# is 1080, the largest number, and the utilization rate of this evacuation passageway is 22.27%. The total number of evacuees from all passages on the third floor is 4133, and the number of evacuees from evacuation channel 7# is 953, with a utilization rate of 23.05%. The total number of people evacuated from all exits on the fourth floor is 3580, and the number evacuated from exits 13#, 14#, 15#, and 16# is more than 650. The total number of evacuees from all the passages on the fifth floor is 2848, and the number from evacuation passage 19# is the largest at 785, and the utilization rate is 27.56%. The total number of evacuees from all passageways on the sixth floor is 1426. The largest number of evacuees from evacuation passageway 24# on the sixth floor is 411, and the utilization rate is 28.82%, followed by evacuation passageway 23# at 373, with a utilization rate of 26.16%. Under normal conditions, the utilization rate of evacuation exits 1#, 7#, 13#, 14#, 15#, 16#, 19#, and 23# is high. The number of evacuees and the utilization rate of stairs in each passage are illustrated in Figure 5.

### 3.2. Analysis of Fire Simulation Results

#### 3.2.1. Fire Scenario Setting

**Figure 6:**
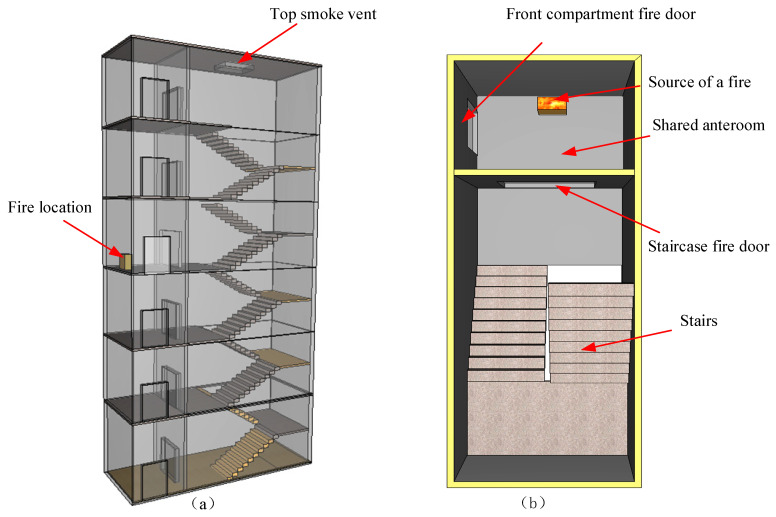
Fire simulation scenario. (**a**) Fire simulation object. (**b**) Staircase structure on the fourth floor.

According to the literature [55], when analyzing the possible fire scenarios, those with low probability of occurrence are not considered; only those with high probability of occurrence or of causing serious consequences are considered. As there are a large number of books on the second to sixth floors of the library, the fire load is large, and the probability of fire occurrence is roughly the same. The scene where fire is likely to cause serious consequences is set as the fire scene. Except for the first floor, the number of people on each floor is more than 600. Under normal circumstances, the use rate of evacuation passages 1#, 7#, 13#, 14#, 15#, 16#, 19#, and 23# is high. Whether these high-crowd evacuation stairs can be used normally has a great impact on the overall evacuation efficiency of the library. Therefore, the fire source is set near the passage with the highest utilization rate. As the passageways 1#, 7#, 13#, 19#, and 23# are located in the same stairwell, to simplify the model these are set as the fire simulation research objects to observe the smoke spread in the case of fire. Due to the large number of reading seats on the fourth floor and above, which are more crowded than other floors, it is most difficult to evacuate in case of fire, so the fire source is located in the front room of the fourth-floor staircase. According to the survey, the stairwell size of the library is 6.8 m × 14 m, with six floors in total; the first floor is 5.4 m high, the other floors are 4.8 m high, the wall thickness is 0.2 m, the size of the fire door is 1.8 m × 2.4 m, and a 1 m^2^ air vent is set at the top of the staircase. The fire source is located at the front room of the fourth floor against the wall, with an area of 1 m × 2 m. A total of 12 thermocouples are set up to measure the temperature of the location and are distributed in the front room of each floor and the center of the stairwell. The top vent and the corresponding doors of each floor are always open during the simulation. The overall material type of the building is set as concrete, with a density of 2280.0 kg/m^3^, a specific heat capacity of 1.04 KJ/(kg·K), and an initial ambient temperature of 20 ℃. The fire simulation scenario is presented in Figure 6.

At present, the steady- and unsteady- state models are commonly used to describe the combustion process of fire [56]. The former is a constant idealized case where the fire heat release rate does not change with time. In reality, the power of the fire source changes with time, and the combustion process will go through four stages: fire, growth, heyday, and decline. For the evacuation of fire personnel, when the fire reaches its peak, the probability of evacuation is very small. Therefore, the setting of fire scenario in the current study mainly considers two stages of fire, fire and growth period, and selects the *t^2^* fire model. The combustibles in the library are mainly books and bookshelves, so wood is selected as the combustible to simulate the burning of bookshelves. The heat release rate is calculated as follows:

(1) $$\dot{Q}=b\cdot t^{2}$$

where $\dot{Q}$ is the heat release rate (KW), *b* is the fire development coefficient (kW/s^2^), and *t*^2^ is the fire development time (s). With reference to the Regulations on Building Smoke Control, the fire power is determined as 5 MW, and the fire development coefficient is 0.04689 kW/s^2^. The time for combustion to reach stability is calculated as 326.55 s. To obtain more comprehensive simulation results, the fire simulation time is set as 900 s.

The fire smoke mainly affects the evacuation of people due to the following factors: temperature, CO concentration, and visibility in the fire site. According to previous research [57], and considering people’s tolerance limit to fire smoke, the following criteria are adopted as fire hazard conditions:

1. Temperature criterion: when the temperature at the safe height (2 m from the ground) reaches above 60 ℃, it is considered to have reached a dangerous state.
2. Criterion of toxic gas concentration: when the CO concentration at the safe height (2 m from the ground) reaches 500 ppm, it is considered to have reached a dangerous state.
3. Visibility criterion: the visibility below the safe height is less than 10 m, where it is considered to have reached a dangerous state.

#### 3.2.2. Fire Simulation Results and Analysis

(1) Analysis of smoke-spreading process

**Figure 7:**
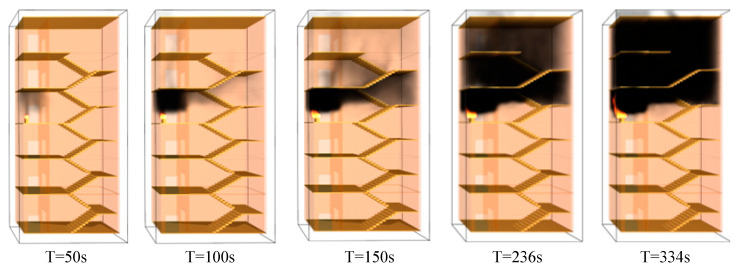
Smoke spread in the stairwell at different times.

This study simulates the fire smoke based on Pyrosim software. From the simulation results, when the fire occurs for 50 s, the smoke in the stairwell is relatively rare, only diffuses in the front room of the fire stairwell, and is not obvious. When the fire occurs for 100 s, a large amount of smoke has gathered in the room before the fire, and a small amount of smoke has spread upward from the front room to the stairwell on the fifth floor. When it reaches 150 s, a large amount of smoke starts to spread to the stairwell on the fifth floor, and a small amount of smoke has already spread to the stairwell on the sixth floor. At 236 s, a large amount of smoke spreads to the stairwell on the sixth floor and the front room, and the evacuation of personnel in this stairwell will be greatly hindered by smoke. At 334 s, the stairwells on the fourth and sixth floors are full of black smoke, and the smoke on the fourth floor continues to spread upward. At this time, the stairwells on the fire floor and above are full of smoke, which no longer meets the conditions for safe evacuation. The spread of smoke in the stairwell at different times is illustrated in Figure 7.

(2) Temperature analysis

**Figure 8:**
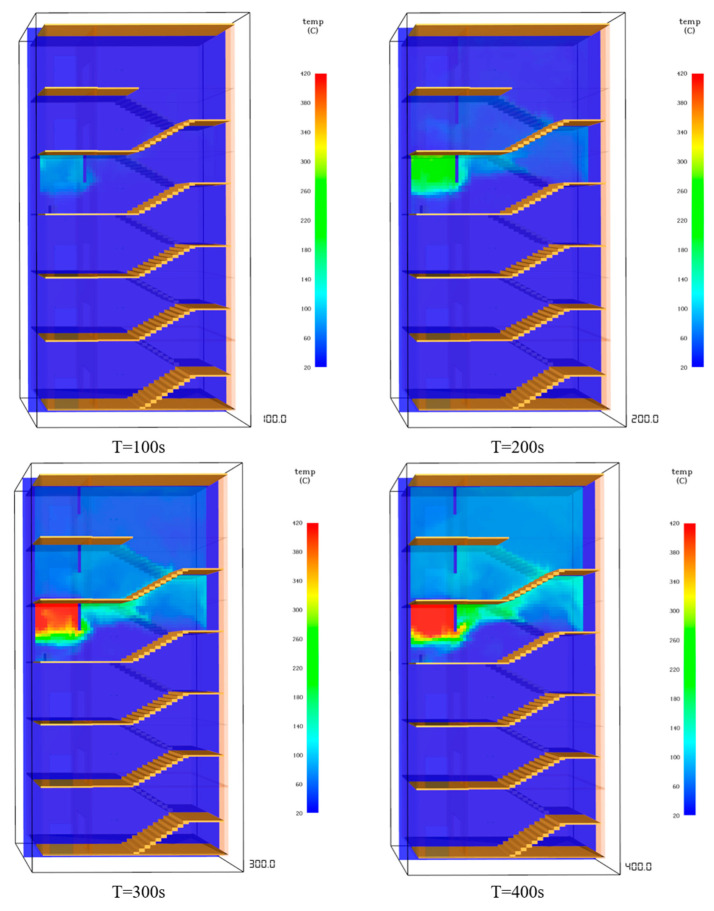
Temperature changes in staircase.

The high-temperature smoke generated by the fire spreads in the stairwell, causing the temperature in the stairwell near the fire source to rise rapidly. The temperature of the stairwell starts to rise from 20 °C, and the temperature of the chamber before the fire rises rapidly after the start of combustion. After 104 s, the temperature exceeds 60 ℃, and the maximum temperature reaches 410 ℃, which seriously threatens the safety of personnel. The temperature of the stairwell on the fire floor rises relatively slowly. The temperature exceeds 60 ℃ in 275 s, reaching a dangerous state. The temperature of the fifth-layer vestibule reaches 60 ℃ at 322 s and keeps rising. The temperature in the stairwell on the fifth floor exceeds 60 ℃ in 327 s. The temperature of the front room on the sixth floor reaches 60 ℃ in 411 s, fluctuating between 80 ℃ and 85 ℃, but the temperature in the stairwell on the sixth floor remains around 20 ℃. The temperature changes in the stairwell when the fire time is 100 s, 200 s, 300 s, and 400 s, as shown in Figure 8.

(3) Visibility analysis

**Figure 9:**
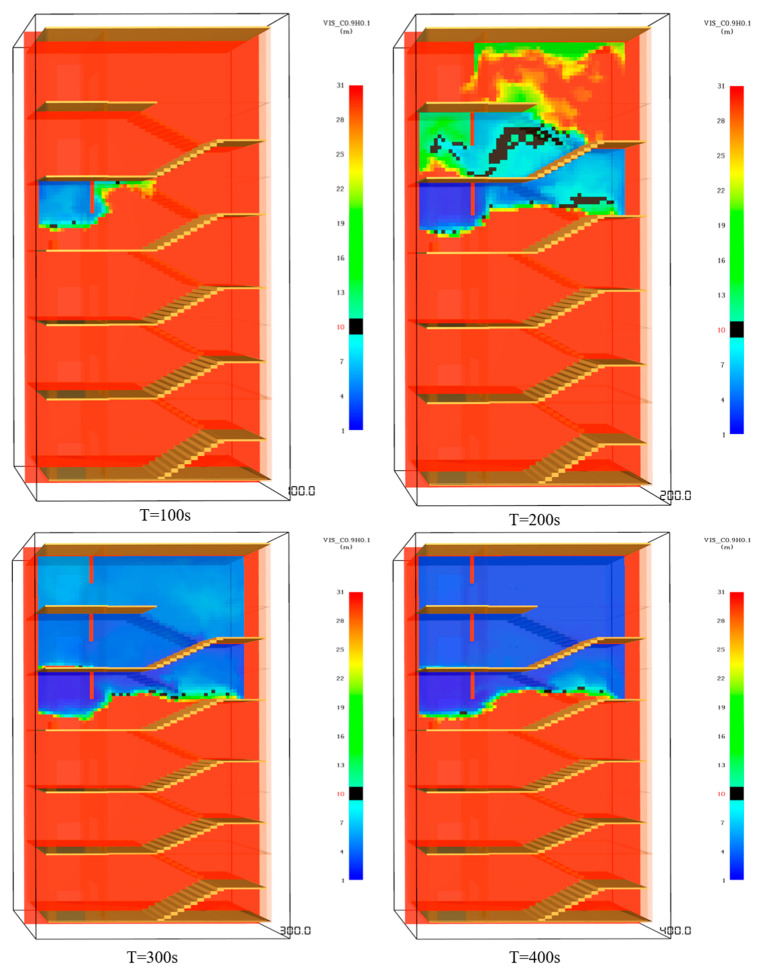
Visibility changes of staircase front room and staircase.

When a fire occurs, the smoke spreads quickly and visibility drops rapidly, which is very unfavorable to the safe evacuation of people. After the front chamber of the fourth and sixth floors is filled with smoke, the visibility rapidly decreases from 30 m to less than 3 m. The visibility of stairwells on the fourth and fifth floors decreases slowly compared with that of the front room, falling below 10 m at 210 s and 240 s, respectively, and below 3 m after 380 s. Due to the smoke vent at the top, the visibility of the stairwell on the sixth floor is always about 30 m. The visibility changes of the front room and stairwell are illustrated in Figure 9.

(4) CO gas-concentration analysis

**Figure 10:**
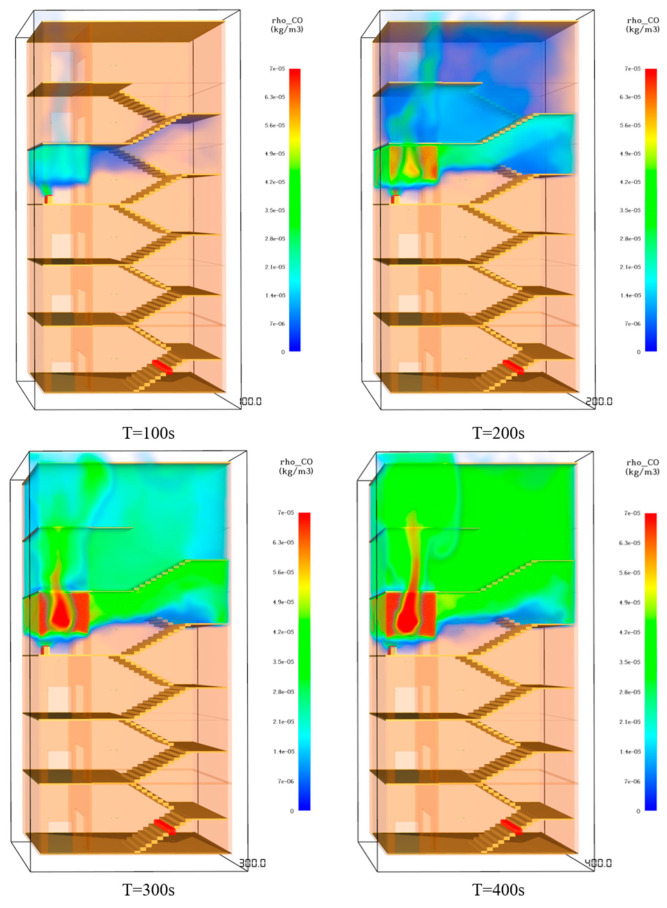
CO gas concentration in the staircase front room and staircase.

In the whole process of simulation, the CO gas concentration in the stairwells and front rooms on the fifth and sixth floors is increasing, and the maximum is 367 ppm, far from reaching 500 ppm, the level of threatening personnel safety. Only the CO concentration in the front room on the fourth floor exceeds 500 ppm after 215 s, and it continues to rise; the maximum is 1400 ppm, seriously endangering human health. The concentration of CO gas in floors one to three is very small, and the harm to personnel can be ignored. The CO gas concentration in the stairwell vestibule and stairwell during the fire spread is illustrated in Figure 10.

Based on the analysis of smoke spread, visibility, temperature, and CO gas-volume fraction nephogram, the allowable safe evacuation time of staircases on each floor is obtained as follows: safe evacuation is available on floors 1–3; the safe evacuation time of the fourth, fifth, and sixth floors is 83 s, 227 s, and 256 s, respectively.

### 3.3. Personnel Evacuation in Case of Fire

According to the fire smoke simulation results, Pathfinder is used to simulate the safe evacuation of people. Stair 13# will be closed after 83 s, Stair 19# after 227 s, and Stair 23# after 256 s. Still considering the most unfavorable conditions, the utilization rate of seats in the library is set to 100%, with 5185 people evacuated; that is, 2173 from the south door, and 3012 from the north door. The shared time is 739 s. The number of evacuees, evacuation time, and stair utilization of each passage are reported in Table 4.

In the evacuation stairs on the second floor, the number of evacuees in Exits 2#, 3#, and 4# is more than 1200, a total of 3773 people are evacuated, and the utilization rate of stairs is 77.79%. The average number of evacuees in exits 1#, 5#, and 6# is 306, and the utilization rate of stairs is only 22.21%. In the evacuation stairs on the third floor, the number of evacuees in exits 8#, 9#, and 10 # is more than 1000, a total of 3274 people are evacuated, and the utilization rate of stairs is 77.69%. The average number of evacuees in exits 7#, 11#, and 12# is 313, and the utilization rate of stairs is 22.31%. The average number of evacuees in evacuation staircases 14#, 15#, and 16# on the fourth floor is 1025, and the utilization rate is 86.07%. The average number of evacuees in evacuation staircases 17# and 18# is 249, and the utilization rate is only 13.93%. In case of fire, the use of evacuation passages is very unreasonable. It is necessary to optimize the library stairs to improve the evacuation efficiency, which is more conducive to the safe evacuation of people.

### 3.4. Evacuation after Man-Made Path Planning

**Figure 11:**
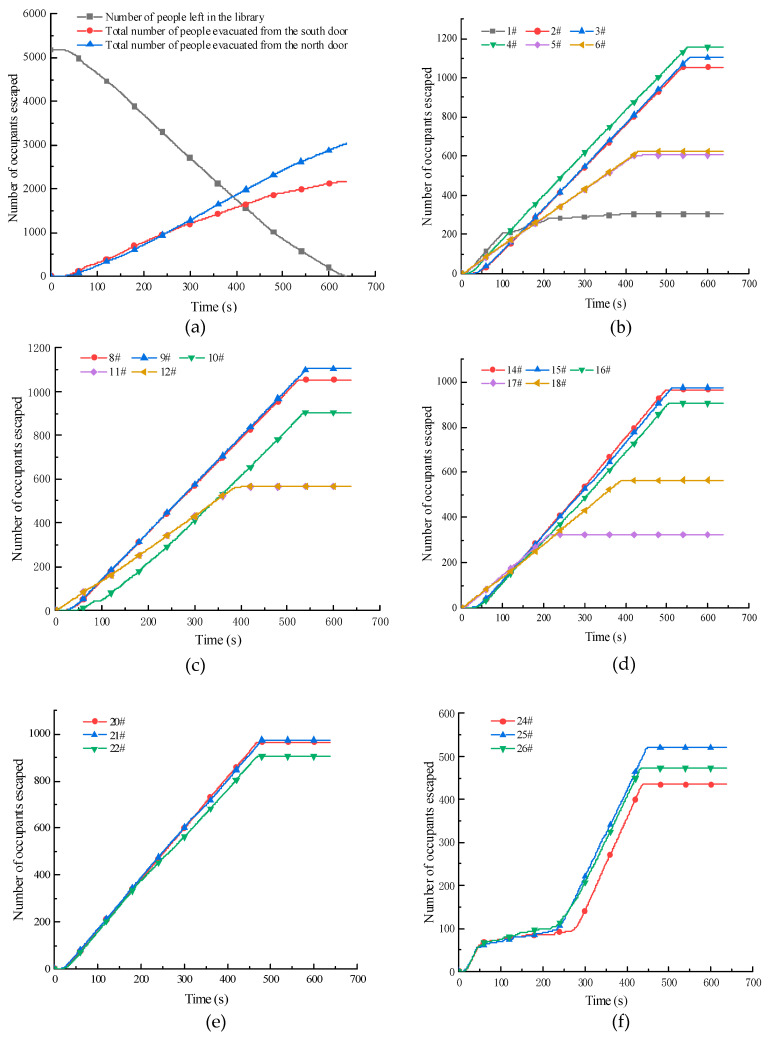
Evacuation of personnel at all levels after optimization. (**a**) Evacuation situation on 1st Floor. (**b**) Evacuation situation on 2nd Floor. (**c**) Evacuation situation on 3rd Floor. (**d**) Evacuation situation on 4th Floor. (**e**) Evacuation situation on 5th Floor. (**f**) Evacuation situation on 6th Floor.

Based on the unreasonable use of the evacuation passageway in the library, this study proposes improving the utilization rate of the evacuation passageway by means of broadcasting guidance, increasing the emergency escape signs, widening the width of stairs, and so on. Pathfinder is used again to simulate the man-made path planning after the fire. The number of evacuees, evacuation time, and stair utilization are presented in Table 5. The number of evacuees in each passage of each floor changes with time, as illustrated in Figure 11.

For channels 1#, 5#, 6#, 7#, 11#, 12#, 17 #, and 18# with fewer people, more people will be directed to these channels through broadcast guidance and additional signs to improve the utilization of stairs. Through proper outlet drainage, the number of people evacuated from evacuation passage 1# increased from 354 to 638, and the utilization rate of stairs increased by 5.49%. The number of people evacuated from passage 5# increased from 334 to 605, and the utilization rate of stairs increased by 5.24%. The number of people evacuated from passage 6# increased from 389 to 475, and the utilization rate of stairs increased by 1.50%. The number of people evacuated from passage 7# increased from 226 to 475, and the utilization rate of stairs increased by 5.75%. The number of people evacuated from evacuation corridors 11# and 12# increased from 357 to 380, and the utilization rate of stairs increased by 0.42%. The number of people evacuated from passage 17# increased from 141 to 323, and the utilization rate of stairs increased by 4.71%. The number of evacuees from passage 18# increased from 357 to 565, and the utilization rate of stairs increased by 5.15%. The increase in the number of evacuees and the utilization rate of evacuation passages are presented in Table 6.

For the passage with a large number of people, the number of evacuees in the evacuation passage can be increased by widening the width of the stairs and drainage. The drainage method can reduce the pressure on the evacuation passage, which is more conducive to the uniform use of stairs and reasonable evacuation of people.

In view of the large flow of people in passages 2#, 3#, 4#, 8#, 9#, 10#, 14#, and 15#, which are relatively congested, evacuation was optimized by widening the width of stairs and appropriate drainage. The results reveal that the number of evacuees in passage 2# is reduced from 1285 to 1024, and the evacuation time is reduced by 108 s; the number in passage 3# is reduced from 1256 to 1052, and the evacuation time is reduced by 110 s; the number in passage 4# is reduced from 1232 to 1178, and the evacuation time is reduced by 77 s; the number in passage 8# is reduced from 1068 to 1042, and the evacuation time is reduced by 108 s; the number in passage 9# is reduced from 1057 to 1052, and evacuation time is reduced by 108 s; the number in passage 10# is reduced from 1149 to 944, and the evacuation time is reduced by 73 s; the number in passage 14# is reduced from 1075 to 966, and the evacuation time is reduced by 93 s; and the number in passage 15# is reduced from 1099 to 975, and the evacuation time is reduced by 88 s. After optimization, the number of evacuees in each floor is more uniform, and the utilization rate of stairs is also more uniform, which avoids waste of resources or overcrowding of evacuation passages. The specific evacuation situation is reported in Table 7.

## 4. Discussion and Limitations

Against the background of rapid economic development, there are more and more high-rise buildings with increasingly complex functions, and there are many fire factors. Some high-rise buildings have more floors or larger areas and are characterized by more fire risk loopholes, loose management systems, more users, and complex internal functions, so they are more prone to major fire accidents [58] When a fire occurs in a high-rise building, it is difficult to evacuate people, which can lead to major casualties. The reasons are related to the characteristics of such buildings: the density of people is high, and safe evacuation is difficult. High-rise buildings have more stories, longer vertical distance, and longer evacuation time [59]. In case of fire, the smoke and fire spread present a vertical characteristic with a fast speed, which will increase the difficulty of safe evacuation, and due to the influence of many factors such as no smoke prevention and power failure, elevators will also be stopped. Therefore, it is of great significance to conduct research on personnel evacuation in public places in the field of public security [60]. However, the previous studies of scholars on the evacuation of public places mainly focus on performance-based fire prevention design, escape path planning based on an algorithm model, the influence of fire smoke spread law on personnel evacuation, and the changes of psychological characteristics and behavior of personnel during evacuation. The previous research on the evacuation of people in public places mainly focuses on the microcosmic aspects, and there is less research on the overall understanding of evacuation in public buildings on the macro level in the case of fire. As a public place for teachers and students to learn, the university library is a typical representative place with dense staff and a large flow of people. This study provides useful suggestions for other public places. Through the investigation of the library, the library model is established according to a 1:1 scale, and the evacuation situation is simulated under the most unfavorable conditions. The evacuation situation after the fire and the man-made route planning are compared and analyzed, and the modified scheme is proposed to effectively solve the problem of low utilization rate of the evacuation channel and long evacuation time. It takes a lot of manpower and financial resources to carry out experiments.

However, the current study has the following limitations. First, human psychological factors are not taken into account. When a fire occurs, everyone’s perception is different. Some people may smell the smell of burning or detect fire burning relatively quickly, but some people will have weak awareness. Second, people’s first reaction to the fire is also inconsistent. Some people will try to leave the fire scene as soon as possible, and some will take firefighting measures immediately. Everyone’s psychological endurance is different. Many people will become frightened and lose their composure when a fire occurs, only wanting to escape quickly. Order is lost, and congestion and stampede accidents are likely to occur, increasing the difficulty of rescue and evacuation. Second, the fire scenario is relatively simple. This is a simplified model, which only considers the location near the highest utilization rate of stairs. In future research, the evacuation of people in different scenarios, such as the reading room, can be considered.

## 5. Conclusions and Suggestions

(1) Based on Revit, Pyrosim, and Pathfinder software, this study conducts an in-depth investigation of evacuation of a university library in the event of a fire. Evacuation exits 1#, 7#, 13#, 19#, and 23# have the highest utilization rate in the library model. The smoke spread, visibility, CO concentration, and temperature at each stairway when a fire occurs near the evacuation exit are also determined. The safe evacuation conditions allowed for people in the stairwells on each floor are as follows: safe evacuation is available on floors 1–3, and the safe evacuation time of the fourth, fifth, and sixth floors is 83 s, 227 s, and 256 s, respectively.
(2) Through the research on the evacuation of library personnel, this study offers optimization suggestions such as increasing broadcast guidance, widening stair width, and proper outlet drainage. In this model, the evacuation time in case of fire is 739.275 s, which is reduced to 638.025 s after man-made route planning, and the evacuation efficiency is increased by 13.67%. The utilization rate of evacuation passages is improved, and the evacuation efficiency is significantly improved, which also provides a new idea and method for the research of evacuation effects in public places and the improvement of facility layouts in the field of public security.
(3) It takes a lot of manpower and financial resources to conduct experiments, and numerical simulation can effectively reduce costs. The current study considers the whole building on the macro level, which is conducive to performance-based fire protection design of building sites. In the early stage of public place design, the cost can be effectively reduced by changing the design according to the feedback of process simulation results. For the built public places, the existing facilities should be reconstructed according to the research results, which can reduce the heavy casualties and economic losses caused by fire and play a preventive role.

## Figures and Tables

**Table 1:** Library evacuation channel number.

Evacuation Outlet	Evacuation Exit Number
1st floor	A, B
2nd floor	1#, 2#, 3#, 4#, 5#, 6#
3rd floor	7#, 8#, 9#, 10#, 11#, 12#
4th floor	13#, 14#, 15#, 16#, 17#, 18#
5th floor	19#, 20#, 21#, 22#
6th floor	23#, 24#, 25#, 26#

**Table 2:** Basic information of each floor.

Floor	1F	2F	3F	4F	5F	6F
Number of seats	335	700	672	632	1420	1426

**Table 3:** Personnel parameter setting.

	Number of People	Shoulder Width (m)	Speed (m/s)
Male	3457	0.5	1.5
Female	1728	0.45	1.28

**Table 4:** Evacuation time and number of evacuees in case of fire.

Escape Route	Number of Evacuees	Evacuation Time (t)	Stair Utilization
1#	354	195	7.30%
2#	1285	640	26.49%
3#	1256	652	25.90%
4#	1232	638	25.40%
5#	334	638	6.89%
6#	389	315	8.02%
7#	226	189	5.36%
8#	1068	628	25.34%
9#	1057	637	25.08%
10#	1149	621	27.27%
11#	357	284	8.47%
12#	357	284	8.47%
14#	1075	593	30.07%
15#	1099	603	30.74%
16#	903	584	25.26%
17#	141	110	3.94%
18#	357	284	9.99%
20#	896	561	32.49%
21#	966	567	35.03%
22#	896	561	32.49%
24#	474	528	33.24%
25#	494	533	34.64%
26#	458	515	32.12%

**Table 5:** Evacuation time and number of evacuees after optimization.

Escape Route	Number of Evacuees	Evacuation Time (t)	Stair Utilization
1#	638	308	12.79%
2#	1042	532	20.88%
3#	1052	542	21.08%
4#	1178	561	23.61%
5#	605	440	12.12%
6#	475	496	9.52%
7#	475	496	11.12%
8#	1042	520	24.39%
9#	1052	529	24.62%
10#	944	548	22.09%
11#	380	482	8.89%
12#	380	482	8.89%
14#	966	500	25.87%
15#	975	515	26.11%
16#	905	506	24.24%
17#	323	213	8.65%
18#	565	503	15.13%
20#	966	471	33.94%
21#	975	481	34.26%
22#	905	473	31.80%
24#	434	438	30.43%
25#	520	449	36.47%
26#	472	434	33.10%

**Table 6:** Increase in number of evacuees and channel utilization rate of each staircase.

Stair No	1#	5#	6#	7#	11#	12#	17#	18#
Increased number of evacuees	284	271	86	249	23	23	182	208
Increase in utilization rate of evacuation routes	5.49%	5.24%	1.50%	5.75%	0.42%	0.42%	4.71%	5.15%

**Table 7:** Reduction of evacuation number and evacuation time of each staircase.

Stair No	2#	3#	4#	8#	9#	10#	14#	15#
Reduced number of evacuees	243	204	54	26	5	205	109	124
Reduction of evacuation time (s)	108	110	77	108	108	73	93	88

## Data Availability

Not applicable.
